# Effect of urolithin A on the improvement of vascular endothelial function depends on the gut microbiota

**DOI:** 10.3389/fnut.2022.1077534

**Published:** 2023-01-05

**Authors:** Yuichiro Nishimoto, Kota Fujisawa, Yuichi Ukawa, Masatake Kudoh, Kazuki Funahashi, Yoshimi Kishimoto, Shinji Fukuda

**Affiliations:** ^1^Metagen Inc., Tsuruoka, Japan; ^2^Department of Life Science and Technology, Tokyo Institute of Technology, Tokyo, Japan; ^3^Healthcare SBU, DAICEL Corporation, Tokyo, Japan; ^4^Healthcare SBU, DAICEL Corporation, Myoko, Japan; ^5^Department of Food Science and Human Nutrition, Faculty of Agriculture, Setsunan University, Hirakata, Japan; ^6^Institute for Advanced Biosciences, Keio University, Tsuruoka, Japan; ^7^Gut Environmental Design Group, Kanagawa Institute of Industrial Science and Technology, Kawasaki, Japan; ^8^Transborder Medical Research Center, University of Tsukuba, Tsukuba, Japan; ^9^Laboratory for Regenerative Microbiology, Juntendo University Graduate School of Medicine, Tokyo, Japan

**Keywords:** urolithin A, gut microbiota, vascular endothelial function, organic acids, flow-mediated vasodilatation

## Abstract

**Background:**

Urolithin A (UA) is a metabolite produced by gut microbiota from ingested ellagic acid. Although the effect of ellagic acid intake on vascular endothelial function (VEF) improvement has been reported, the effect of UA intake on VEF improvement remains obscure. In addition, UA has been reported to improve the intestinal barrier function, and UA may have improved VEF by gut microbiome alteration.

**Objective:**

In this study, we conducted a clinical trial to explore and analyze the effects of UA intake on vascular endothelial function (VEF) and characteristics of the intestinal environment, such as gut microbiome profiling and organic acid composition.

**Methods:**

A placebo-controlled, randomized, double-blinded, parallel group trial was conducted on participants who could metabolize small amounts of UA from ellagic acid (non-UA producers) and had relatively poor VEF. VEF was assessed using the flow-mediated vasodilatation (FMD) score. Participants were administered placebo, UA 10 mg/day, or UA 50 mg/day for 12 weeks. FMD was measured and fecal samples were collected at 0, 4, 8, and 12 weeks of treatment. Gut microbiome analysis and organic acid level measurements were performed to evaluate the effects of UA intake on the intestinal environment. This clinical trial is publicly registered at the UMIN-CTR, trial number: UMIN000042014.

**Results:**

The gut microbiota of the UA 50 mg/day group showed a significant increase in alpha diversity (Faith’s phylogenetic diversity). Four and nine microbial genera were significantly altered in the UA 10 mg/day and UA 50 mg/day groups, respectively (*p* < 0.05, not corrected). Participants whose FMD scores improved with UA intake had poor baseline FMD values as well as a low Bacillota/Bacteroidota ratio.

**Conclusion:**

Urolithin A intake alters the gut microbiota and improves their alpha diversity. In addition, the effect of UA on VEF correlated with the individual gut microbiota. Our results have practical implications for a new approach to providing healthcare that focuses on intestinal environment-based diet therapy.

## 1. Introduction

Cardiovascular diseases are the leading cause of death worldwide, having caused 17.8 million deaths in 2019. This figure is expected to increase to 23 million by 2030 (1, 2). The symptoms of cardiovascular diseases worsen with age due to the deterioration of vascular endothelial function (VEF) (3). Degradation of VEF also has been reported to be associated with CVD events such as cardiac death, myocardial infarction and stroke, type 2 diabetes, chronic kidney disease, and mild cognitive impairment (4–7).

The gut microbiota and their derived metabolites influence VEF. For example, the synbiotic intake of *Bifidobacterium animalis* and arginine improves VEF *via* gut microbiota-derived polyamines (8). Urolithin A (UA) is a microbiota-derived metabolite produced from dietary ellagic acid present in certain foods, including pomegranates, berries, and walnuts. UA improves mitochondrial function by activating mitochondrial recycling (9). Other benefits include improved exercise performance and enhancement of gut barrier integrity (10, 11). A previous study reported that UA inhibits intestinal permeability by activating the aryl hydrocarbon receptor-nuclear factor erythroid 2-related factor 2 pathway (10). In addition, there is a report that a single dose of raspberry containing ellagic acid, a precursor of UA, improved VEF (12). Therefore, as a microbiota-derived metabolite, UA may alter the intestinal environment, thereby improving the VEF.

The UA-producing ability varies among individuals because of differences in the gut microbiota involved in ellagic acid metabolism. In the previous studies, some gut microbes such as *Gordonibacter urolithinfaciens* DSM 27213^T^ = CCUG 64261^T^, *Gordonibacter pamelaeae* DSM 19378^T^ = CCUG 55131^T^, *Ellagibacter isourolithinifaciens* DSM 104140^T^ = CCUG 70284^T^, *Bifidobacterium pseudocatenulatum* INIA P815, and *Enterococcus faecium* FUA027 have been reported to produce urolithins from ellagic acid (13–16). Among them, *B. pseudocatenulatum* INIA P815 and *Enterococcus faecium* FUA027 showed urolithin A-producing ability (15, 16). Despite this, not all urolithin-producing bacteria are known. Approximately 40% of human individuals harvest a microbiome capable of producing UA (17). Further, UA-producers have a high Bacillota/Bacteroidota ratio. In previous studies, there were individual differences in the effects of oral intake of foods and drugs, which depended on the gut microbiota (18, 19). Thus, it is possible that the efficacy of UA supplementation depends on an individual’s intestinal environmental features (gut microbiota and metabolites).

In this study, we conducted a clinical trial on UA non-producers with relatively poor VEF scores to examine the effects of UA on gut microbiota, organic acids, and VEF. Furthermore, we analyzed the correlation between intestinal environmental features and UA-induced improvement in flow-mediated vasodilatation (FMD) scores.

## 2. Materials and methods

### 2.1. Clinical trial

**FIGURE 1:**
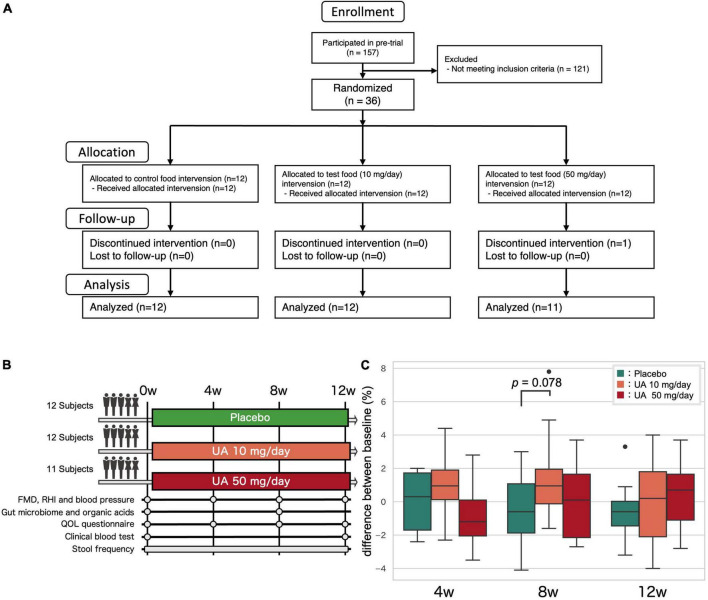
Clinical trial overview. **(A)** Clinical trial flow diagram. **(B)** Overview of the clinical trial. Fecal samples were collected at the 0, 4, 8, and 12 weeks and analyzed at 0 and 8 weeks. **(C)** The effect of UA on the FMD. *P*-values for the groups that showed an increasing trend compared to those for the placebo group (Wilcoxon’s rank-sum test). UA, urolithin A; FMD, flow-mediated vasodilatation; RHI, reactive hyperemia index; QOL, quality of life.

A randomized, double-blinded, placebo-controlled, three-way parallel-group study was conducted on Japanese individuals for 12 weeks (Figure 1A and Supplementary Table 1). Consent was obtained from the participants, and the study adhered to the Helsinki Declaration and Ethical Guidelines on Epidemiological Research in Japan referring to cases concerning standards for clinical trials of drugs. The experimental groups were provided capsules containing 10 or 50 mg of UA, and the control group was provided UA-free capsules; these were to be consumed daily throughout the 12 weeks. The primary outcome was FMD score using UNEXEF18VG (UNEX Corporation, Nagoya, Japan). The secondary outcomes were blood pressure (systolic and diastolic), reactive hyperemia index (RHI) using Endo-PAT2000 (Doctor Planets Ltd., Hyogo, Japan), gut microbiome, organic acids, stool data (stool days, frequency, amount, property, color, feeling of residual, smell, and incidence of abdominal pain during defecation), quality of life questionnaire responses, and clinical blood tests. During the trial, FMD, blood pressure, quality of life questionnaire data, and fecal samples were collected at 0, 4, 8, and 12 weeks of the dietary intervention period. The stool samples were frozen at –20°C until analysis. Depending on the primary outcome results, further microbiome and organic acid analyses were performed on the stool samples from weeks 0 to 8. Blood samples were collected at 0 and 12 weeks.

This trial was conducted with the approval of the Clinical Trial Ethics Review Committee of the Chiyoda Paramedical Care Clinic (publicly registered at UMIN-CTR, trial number: UMIN000042014^1^). The inclusion criteria were as follows:

(1) Male or female aged between 40 and 65 years.
(2) Individuals whose FMD was below 7.0%.
(3) Individuals who are UA non-producer or low producer.
(4) Individuals who could understand the study procedure, agreed to participate in the study, and provided informed consent prior to the study.

The exclusion criteria were as follows:

(1) Individuals who were currently receiving medication.
(2) Individuals who planned to or already had taken medication (e.g., drug for intestinal disorder, antihypertensive, laxative, or antibiotic) a month prior to the trial, which would have affected the results.
(3) Individuals who regularly consumed food for specified health uses, food with function claims, supplements, and/or health foods that would affect the trial results, more than three times per week.
(4) Individuals who were breastfeeding, pregnant, or are planning pregnancy.
(5) Individuals who have current medical history/anamnesis of severe cardiac, hepatic, renal, or digestive disease.
(6) Individuals with high alcohol consumption.
(7) Individuals with a history of smoking. However, if the individual had quit smoking for more than a month prior to trial, the individual could be included.
(8) Individuals with irregular lifestyle and diet.
(9) Individuals who have allergies to food or medication.
(10) Individuals who are currently participating, have participated in other studies on medicine or food a month prior to the trial, or participated in other clinical trials after agreeing to participate in this trial.
(11) Individuals who had donated more than 200 ml of blood and/or blood components a month prior to the trial.
(12) Males who had donated more than 400 ml of blood and/or blood components 3 months prior to the trial.
(13) Females who had donated more than 400 ml of blood and/or blood components 4 months prior to the trial.
(14) Males who will receive over 1,200 ml of blood and/or blood components, when the sampling amounts within the last 12 months are added to the planned sampling amounts of this study.
(15) Females who will receive over 800 ml of blood and/or blood components when the sampling amounts within the last 12 months are added to the planned sampling amounts of this study.
(16) Others who were deemed ineligible by the principal investigator or sub-investigator.

A total of 157 individuals participated in the pre-trial, from which 36 were selected for the main trial based on the inclusion and exclusion criteria. To select UA non-producers or low-producers, the participants were instructed to consume a commercially available food containing ellagic acid (pomegranate seed extract, DHC, Tokyo, Japan) before sleeping on the night of the pre-test, and the first urine sample was collected early the next day, which was analyzed to measure the urinary UA. Those with urinary creatinine-corrected UA less than 1.00 nM/mg were categorized as UA non-producers or low producers. Among the initial 157 participants, 73, 14, and 70 participants were designated as UA non-producers, UA low-producers, and UA-producers, respectively. Randomization was performed using the block-stratified randomization method with the participant assignment manager. Participants who met the inclusion criteria were assigned to three groups (P, Q, and R) by stratification, while accounting for age, sex, pre-tested FMD, and blood pressure values. Subsequently, the symbols “P,” “Q”, and “R” were randomly assigned to each group of participants. A test food assignment table with the test food symbol and participant identification code was prepared. Immediately after assignment to the test food, the table was sealed and concealed by the participant assignment manager. The table was only disclosed to the test analyst, investigator, and test-sharing doctor after data collection. A total of 36 participants were included in the main trial. One participant dropped out due to refusal to visit the hospital. Hence, 35 participants completed the trial.

### 2.2. Gut microbiome analysis

The extraction and measurement of DNA and organic acids from fecal samples were performed as previously described (20). Briefly, the samples were lyophilized using a VD-800R lyophilizer (TAITEC Corp., Saitama, Japan) for at least 24 h. The lyophilized stool samples were then separated for DNA and metabolite extraction. After DNA extraction, the V1–V2 variable region of the 16S rRNA gene was amplified using the bacterial universal primers 27F-mod (5′-AGRGT TTGATYMTGGCTCAG-3′) and 338R (5′-TGCTGCCTCCC GTAGGAGT-3′) with Tks Gflex DNA Polymerase (Takara Bio Inc., Shiga, Japan) (21). Amplicon DNA was sequenced using MiSeq (Illumina, USA), according to the manufacturer’s protocol. All 16S rRNA amplicon sequence files generated in this study are available in the DRA of the DNA Data Bank of Japan (DRA accession number: DRA014918).

### 2.3. Organic acid level measurement

Metabolites were extracted from feces using the following procedure. Freeze-dried feces were disrupted with 3.0 mm zirconia beads by vigorous shaking (1,500 × *g* for 10 min) using a Shake Master (Biomedical Science, Tokyo, Japan). Fecal samples (10 mg) were suspended in 1,000 μl of internal standard (crotonic acid), followed by 500 μl of concentrated HCl and 2,000 μl of ether added to each tube. The tubes were then vigorously shaken (1,500 × *g* for 10 min) using a Shake Master and centrifuged at 10,000 × g for 10 min. After centrifugation, 80 μl of the top ether layer was collected and added to an Agilent crimp-cap vial containing 16 μl of N-tert-butyldimethylsilyl N-methyltrifluoroacetamide for gas chromatography. The vials were capped with a crimp-top natural rubber/polytetrafluoroethylene seal type seven aluminum silver 11 mm chromacol cap and sealed using a crimper. After tapping and mixing, the vials were tightly sealed with parafilm and heated in a water bath at 80^°^C for 20 min. The capped vials were then left overnight at room temperature for derivatization. Calibration was achieved using standard solutions of derivatized formic, acetic, propionic, isobutyric, butyric, isovaleric, valeric, lactic, and succinic acids, as described for the test samples. The final concentrations of each standard were 50, 30, 20, 10, 5, 1, 0.5, 0.1, 0.05, and 0.01 mM. The derivatized samples were run through a 7,890 series GC–MS system (Agilent Technologies, CA, USA) fitted with a DB-5ms column (0.25 mm × 30 m × 0.25 μm; Agilent Technologies). Helium was used as the carrier gas and delivered at a flow rate of 1.2 ml/min. The head pressure was set at 10.5 psi with a split ratio of 100:1. The injector, ion source, quadrupole mass spectrometer, and transfer line were set to 250, 230, 150, and 260^°^C, respectively. Two microliters of each sample were injected with a run time of 30 min. The measurement was controlled using the Agilent MassHunter Workstation Data Acquisition software (version 10.0, Agilent Technologies), and the obtained data were analyzed using the Agilent MassHunter Quantitative Analysis software (version 10.1, Agilent Technologies). Organic acid data are presented in Supplementary Table 2.

### 2.4. Bioinformatics and statistical analysis

For 16S rRNA gene-based microbiome analysis, QIIME2 (version 2019.10) was used (22). Primer bases were trimmed using cutadapt (option: –p-discard-untrimmed) (23). Sequence data were processed using the DADA2 pipeline for quality filtering and denoising (options: –p-trunc-len-f 230 –p-trunc-len-r 130) (24). The contamination of human genome was checked by mapping the filtered output sequences, and no contamination were found. The filtered output sequences were assigned to taxa using the “qiime feature-classifier classify-sklearn” command with the default parameters (25). Silva SSU Ref Nr 99 (version 132) was used as the reference database for taxonomic assignment. Alpha and beta diversities were calculated using “qiime phylogeny align-to-tree-mafft-fasttree” and “qiime diversity core-metrics-phylogenetic” commands with the sampling depth to the lowest read numbers. The microbiome data are presented in Supplementary Table 3.

All statistical analyses were performed using Python scripts (version 3.7.6). Multidimensional scaling was performed using unweighted/weighted UniFrac distance calculated from the microbiome amplicon sequence variants (ASVs) data (scikit-learn version 0.20.0). For pairwise comparison of bacteria, the Wilcoxon signed-rank test with Benjamini–Hochberg false discovery rate correction was used (scipy version 1.5.2 and statsmodels version 0.10.0, respectively). A trend test (Jonckheere-Terpstra test) was also performed to identify consistent changes in bacteria between the placebo, UA 10 mg/day and UA 50 mg/day groups (scipy version 1.5.2). Bacteria with mean relative abundance below 0.001 were excluded from the comparison. In the comparison of organic acids, two-way analysis of variance were used (statsmodels version 0.10.0). In this study, the test food effect size for each participant was defined as the responder score and was used to determine whether the effects depended on individual basal characteristics. The response score was defined as the value of each individual after 8 weeks of intake minus the baseline. In the responder feature analysis, the Spearman rank correlation coefficient and the test for no correlation were used (scipy version 1.5.2).

## 3. Results

### 3.1. Effect of UA on the gut microbiome profile

A placebo-controlled, randomized, double-blinded, parallel group trial was conducted on 36 Japanese UA non-producers or low-producers (Figures 1A, B). Baseline (0 weeks) characteristics were similar in all three groups, but FMD scores tended to be slightly higher in the 50 mg/day group (Placebo group: 5.9 ± 2.0%, 10 mg/day group: 4.7 ± 2.2%, 50 mg/day group: 6.6 ± 2.3%; *p*-value in Kruskal-Wallis test: 0.148; Supplementary Table 4). One participant dropped out due to refusal to visit the hospital and 35 participants completed the trial. Effects of UA intake other than those on gut microbiota and metabolites have been reported in a previous study (26).

**FIGURE 2:**
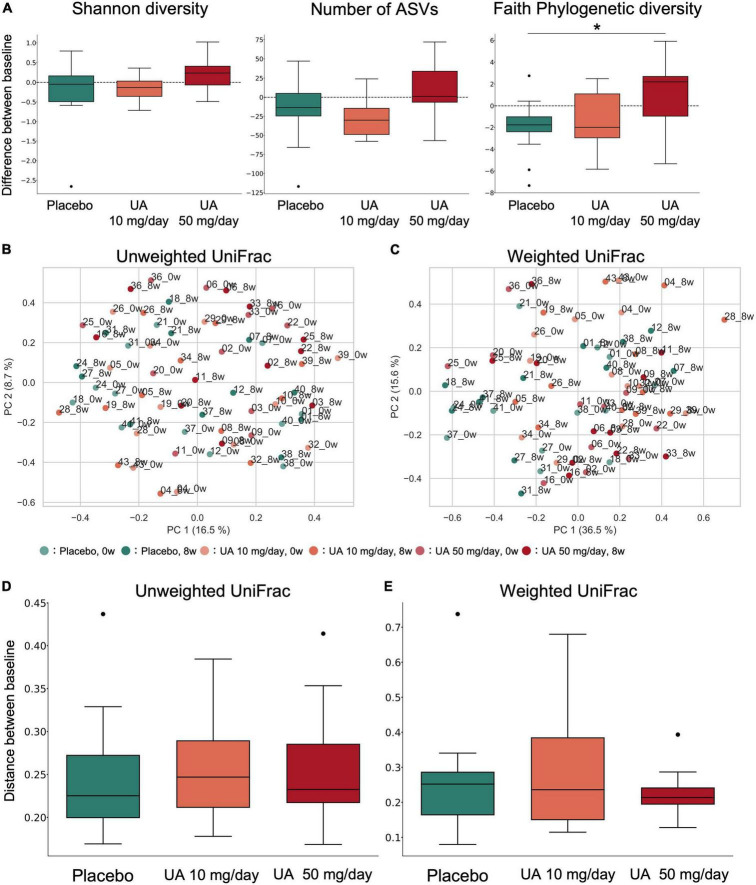
α- and β-diversity of the gut microbiota. **(A)** Box plot of alpha diversity indices of gut microbiota profiles (left: Shannon diversity; middle: number of ASVs; right: Faith’s phylogenetic diversity). *p < 0.05; Dunn’s tests. **(B,C)** Principal coordinate analysis was performed on the samples’ **(B)** unweighted UniFrac distance or **(C)** weighted UniFrac distance. This percentage represents the contribution ratio. **(D,E)** The **(D)** unweighted UniFrac distance and **(E)** weighted UniFrac distance were calculated for each participant between 0 and 8 weeks of UA intake and are shown as box plots for each group. Kruskal–Wallis tests were performed between groups; however, no significant differences were detected. UA, urolithin A; ASV, amplicon sequence variant.

To investigate the effect of UA intake on the participants’ gut microbiota, we performed 16S rRNA gene-based microbiome analysis at 0 and 8 weeks following UA intake. No significant improvements in all VEF score (FMD, RHI, systolic blood pressure, and diastolic blood pressure) were observed (Figure 1C and Supplementary Table 5). As there is a known link between the alpha diversity of the gut microbiota and host metabolism and disease, the alpha diversity of the placebo and UA groups was compared. Shannon diversity, number of bacteria (ASV count), and Faith’s phylogenetic diversity (Faith’s PD) were calculated as alpha diversity indices. We found significant differences in Faith’s PD between the placebo and UA 50 mg/day groups (Dunn’s test; Figure 2A). A beta diversity analysis of the gut microbiome profile was also performed (Figures 2B, C). Furthermore, unweighted and weighted UniFrac distances before and after UA intake were calculated for each treatment group (Figures 2D, E). The results showed no significant differences among the three groups, suggesting that intake of 10 and 50 mg/day of UA had no significant effect on the overall gut microbiome profiles.

### 3.2. Effect of UA on gut microbes and organic acids

**FIGURE 3:**
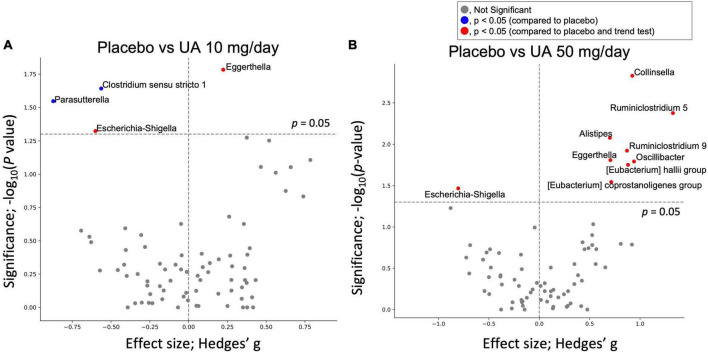
Effect of UA on gut bacteria. Volcano plot of the effect of UA intake on gut bacteria; X-axis represents the Hedges’ g compared with the placebo group. Y-axis is the logarithm of the statistical test’s *p*-value (Wilcoxon signed-rank test). **(A)** Compares the placebo group with UA 10 mg/day group, and **(B)** compares the placebo group with UA 50 mg/day group. UA, urolithin A.

Gut microbial genera with differential abundances between the placebo and UA groups were detected using the Wilcoxon rank-sum test. Compared with the placebo group, four and nine genera were significantly altered in the UA 10 mg/day and UA 50 mg/day groups, respectively (Figures 3A, B). However, the results were not significant after multiple test corrections (false discovery rate-corrected *q*-value >0.10). In addition, a trend test revealed consistent changes in bacteria in the placebo, UA 10 mg/day, and UA 50 mg/day groups, with changes in the 50 mg/day group consistent with those in the three groups (Figure 3B). In addition, the changes in decreasing of *Escherichia*-*Shigella* and increasing of *Eggerthella* were consistent in the 10 mg/day group and 50 mg/day group. To further investigate the effects of UA intake on the intestinal environment, the similar analysis was performed using organic acids. Differences among placebo, UA 10 mg/day, and UA 50 mg/day groups were detected for formic acid and propionic acid (*p* < 0.05, Table 1).

**TABLE 1:** Amount of organic acids in each group.

(μmol/g)	Placebo				UA 10 mg/day				UA 50 mg/day				*P*-value		
	0 week		8 weeks		0 week		8 weeks		0 week		8 weeks		Timepoint	Group	Timepoint and group
Formic acid	2.77	(0.04)	2.76	(0.11)	2.84	(0.07)	2.14	(0.05)	3.15	(0.08)	3.75	(0.23)	0.839	0.019	0.161
Acetic acid	146.3	(7.53)	199.1	(9.53)	143.3	(6.18)	157.8	(6.87)	134.5	(6.45)	149.1	(9.56)	0.142	0.384	0.624
Propionic acid	69.7	(4.29)	86.56	(3.39)	52.13	(2.03)	57.44	(2.64)	53.67	(1.89)	56.65	(3.81)	0.260	0.018	0.721
Isobutyric acid	3.55	(0.15)	3.4	(0.19)	3.68	(0.16)	3.43	(0.21)	2.91	(0.14)	3.53	(0.17)	0.897	0.793	0.652
Butyric acid	30.28	(1.62)	35.26	(1.95)	31.93	(1.04)	38.14	(2.58)	27.09	(1.56)	29.55	(1.78)	0.293	0.452	0.939
Isovaleric acid	2.85	(0.15)	2.65	(0.16)	3.02	(0.16)	2.82	(0.22)	2.52	(0.15)	3.02	(0.16)	0.957	0.926	0.714
Valeric acid	4.7	(0.39)	5.05	(0.54)	4.31	(0.22)	5.7	(0.38)	7.03	(0.56)	6.66	(0.58)	0.665	0.281	0.809
Lactic acid	0.31	(0.01)	1.3	(0.25)	0.37	(0.02)	0.45	(0.02)	1.28	(0.31)	0.47	(0.03)	0.775	0.570	0.174
Succinic acid	1.63	(0.26)	20.68	(4.55)	7.00	(2.12)	3.94	(0.69)	17.18	(5.41)	19.87	(3.75)	0.431	0.421	0.498

Values are presented as mean (standard deviation). *P*-value was calculated by two-way analysis of variance. UA, urolithin A.

### 3.3. Correlation analysis between the FMD score, gut microbiome, and organic acids before and after UA intake

**FIGURE 4:**
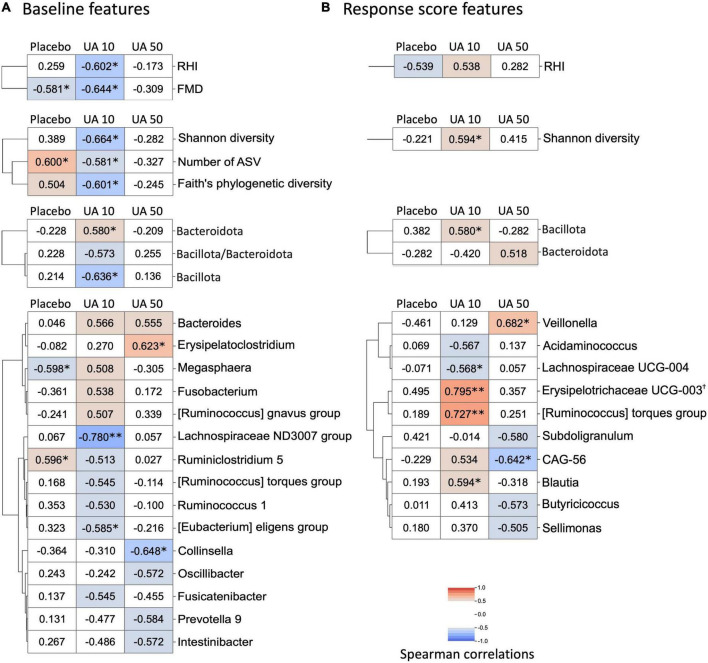
Heatmap of correlation analysis of FMD response score. **(A)** Baseline features that correlated with FMD response score. Correlation analysis between FMD response scores and baseline gut microbes, organic acids, and VEF scores was performed. Correlations with statistical significance or with absolute Spearman’s correlation coefficient larger than 0.50 in either treatment group are shown in the heatmap (*p < 0.05; ^**^p < 0.01, Spearman’s correlation test). Row clustering using the Ward method was performed for each of the four data categories (VEF score, alpha-diversity, phylum, and genus). When FDR is corrected, none of the correlations are significant (FDR corrected *q*-value >0.10). **(B)** Correlation analysis between FMD response score and gut microbes, organic acids, and VEF response scores. Correlations with statistical significance or with absolute Spearman’s correlation coefficient larger than 0.50 in either treatment group were shown in the heatmap (*p < 0.05; ^**^p < 0.01, Spearman’s correlation test; ^†^FDR corrected *q*-value <0.10). Row clustering using the Ward method was performed for each of the four data categories (VEF score, alpha-diversity, phylum, and genus). UA, urolithin A; UA 10, UA 10 mg/day; UA 50, UA 50 mg/day; VEF, vascular endothelial function; FMD, flow-mediated vasodilatation; RHI, reactive hyperemia index; FDR, false discoversy rate.

A correlation analysis was performed to investigate the relationship between the participants’ response to UA intake and their clinical features, such as gut microbiota, organic acids, and VEF score. We defined the difference in FMD scores before and after UA intake as FMD response scores, and responses for other clinical features were defined in a similar fashion. Correlation analysis between FMD response score and baseline clinical features showed that baseline FMD values were negatively correlated with FMD response scores for participants in the placebo and UA 10 mg/day groups (Figure 4A). In the UA 10 mg/day group, FMD response scores correlated with baseline RHI values. Several correlations were observed between the FMD response scores and the baseline gut microbiome features of the UA 10 mg/day group, such as the FMD response score’s positive correlation with Bacteroidota as well as negative correlation with Bacillota and all calculated alpha diversity indices (Shannon diversity, number of ASVs, and Faith’s PD). Regarding the Bacteroidota genera, especially *Bacteroides* and *Prevotella* 9, baseline *Bacteroides* showed a positive correlation with the FMD response score, whereas *Prevotella* 9 showed a negative correlation (Figure 4A). None of the organic acids were significantly correlated. Finally, we analyzed the correlation between the FMD and clinical feature response scores (Figure 4B). The results showed that the FMD response score and the Bacillota, and Shannon diversity response scores had a significant positive correlation in the UA 10 mg/day group, suggesting that these features could be UA responder markers (Figure 4B). After FDR correction, only the correlation between Erysipelotrichaceae UCG-003 response score and FMD response score was significant in the UA 10 mg/day group (Figure 4B, *q*-value < 0.10).

## 4. Discussion

A trend toward FMD score improvement was observed at 8 weeks after treatment of UA 10 mg/day group (*p* = 0.078), suggesting that UA intake potentially improves VEF. However, this result did not account for the fact that multiple groups and time periods were involved in this study because of the small sample size. Therefore, a larger interventional study is necessary to demonstrate the effect of UA intake on VEF. Although the UA 50 mg/day group was assumed to be more effective, there was no difference at any time point in this clinical trial. This may be due to higher FMD scores at week 0 in the UA 50 mg/day group (Supplementary Table 4), given that the FMD response score (difference in FMD scores before and after UA intake) and baseline FMD scores were negatively correlated for participants in the placebo and UA 10 mg/day groups (Figure 4A).

Alpha diversity of the human gut microbiome has been linked to host metabolism and diseases. Upon analysis, Faith’s PD of the UA 50 mg/day group was significantly higher than that of the placebo group. As other alpha diversity indices did not show significant differences, it is likely that gut microbes with a low relative abundance and distant phylogenetic distance were enriched in the gut microbiota of the UA 50 mg/day group. A previous study reported that Western diets increased the number of obese individual’s with a decrease in Faith’s PD (27). In addition, negative correlations between inflammatory scores and Faith’s PD in mice have been reported (28). Although previous studies have not proven a causal relationship between Faith’s PD and these phenotypes, higher Faith’s PD may lead to the maintenance of intestinal homeostasis and prevention of obesity. Beta diversity analysis of the gut microbiome showed no significant differences in gut microbiome in either UA intake group compared to that in the placebo group, suggesting that UA had no significant effect on the overall gut microbiome profiles.

Regarding the individual microbes, although no significant differences were detected after FDR correction, four microbial genera in the UA 10 mg/day group and nine genera in the UA 50 mg/day group showed significant differences compared with those in the placebo group. Of these, a trend test revealed consistent changes in bacteria in the placebo, UA 10 mg/day, and UA 50 mg/day groups, with changes in the 50 mg/day group consistent with those in the three groups (Figure 3B). The relative abundance of *Ruminiclostridium* 5, (*Eubacterium*) *coprostanoligenes* group, and (*Eubacterium*) *hallii* group was significantly higher in the UA 50 mg/day group than that in the placebo group. *Ruminiclostridium* 5 enrichment restored circadian rhythm disturbances and decreased body temperature and sleep disturbances in rats (29). In addition, insulin activity in a type II diabetes mouse model was restored upon oral administration of *Eubacterium hallii* (30). Furthermore, the *Eubacterium coprostanoligenes* was found to lower blood cholesterol by converting cholesterol to coprostanol, a less absorbable form (31). These results suggest that UA 50 mg/day intake may induce lower blood cholesterol levels, improve insulin sensitivity, and regulate the circadian rhythm. In the previous studies, UA also suppresses intestinal permeability *via* the aryl hydrocarbon receptor-nuclear factor erythroid 2-related factor 2 pathway (10) and reduces intestinal inflammation by suppressing the NF-κB (32); Inclusion criteria in this study were individuals with relatively poor VEF, who may also have relatively more intestinal inflammation compared to healthy individuals. Therefore, it is possible that suppression of intestinal inflammation may have an indirect effect on the intestinal microbiota. Intestinal organic acid analysis show significant differences only in two organic acid such as formic acid and propionic acid among placebo, UA 10 mg/day, and UA 50 mg/day groups (Table 1). Formic acid was higher in the UA 50 mg/day group at baseline and increased on average after intake, suggesting that UA intake may have affected formic acid-producing bacteria and increased formic acid. In addition, propionic acid was higher in the placebo group for both 0 and 8 weeks. This may might have influenced the differences between three groups. Hence, it is possible that while UA had some effects on the gut microbiota, it may have a minor effect on intestinal metabolites.

Differences in individual responses to the oral intake of food and drugs have been observed, with studies showing a link with gut microbiota (18, 19). In the placebo and UA 10 mg/day groups, improved FMD response scores were observed in the participants with lower FMD baseline values. As the placebo group also showed same trend, it is possible that there may be cyclic variation in FMD values. In a previous study, the FMD values were lower in winter than those in the other seasons (33). Whether this variation depends on the initial values remains unknown; however, this should be considered in subsequent analyses. A previous study reported that UA non-producers have low ratio of Bacillota (old name, Firmicutes) to Bacteroidota (old name, Bacteroidetes), and high alpha diversity in their intestines (17). From the correlation analysis between FMD response score and baseline clinical features, we found that the baseline Bacteroidota of the UA 10 mg/day group had a positive correlation with FMD response score, while Bacillota and alpha diversity had a negative correlation. These observations are consistent with the observations obtained for gut microbiota of UA non-producers in a previous study (17). In this study, UA non-producers were selected based on their urinary UA concentration at the time of ellagic acid intake in the pre-trial. Stronger characteristics of UA non-producers in the intestinal environment may be correlated with the effect of UA on improving VEF. As a regular diet generally contains some ellagic acid, it is assumed that supplementation will have a stronger effect on UA non-producers. Further, the FMD response score correlated with increase in relative abundance of Bacillota and Shannon’s diversity, thus becoming associated with the gut microbiota of UA producers. UA intake may cause positive feedback on gut microbial UA production by increasing the number of bacteria that can produce UA from ellagic acid. Some bacteria, such as *Gordonibacter pamelaeae* and *G. urolithinfaciens*, can produce urolithin C from ellagic acid, and *Clostridium bolteae*, *C. asparagiforme*, and *C. citroniae* can produce UA from urolithin C (34). Regarding the known UA-producing bacteria, *Gordonibacter* and *Lachnoclostridium*, based on the database used in this study, there was no significant correlation between the baseline and response scores (Figure 4). Assuming that the subject’s gut microbiota is approaching the gut microbiota of a UA producer, it is possible that the major UA-producing bacteria in the human gut may be neither *Gordonibacter* nor *Lachnoclostridium*. In addition, *Lachnoclostridium* were detected in all samples; therefore, it is possible that species other than UA-producing bacteria were detected (Supplementary Table 3). *Gordonibacter* was rarely detected in this study (placebo group: four participants at 0 weeks and two participants at 8 weeks; UA 10 mg/day intake group: one participant at 0 weeks and zero participants at 8 weeks; UA 50 mg/day intake group: zero participants at 0 weeks and three participants at 8 weeks). *Gordonibacter* is a minor gut microbe that may be below the quantification limit. Therefore, a quantitative polymerase chain reaction test is necessary to evaluate the abundance of this genus.

The limitation of this study was the small sample size (approximately 12 participants per group). Although this study used two dosages, the results were inconsistent. Thus, a large interventional study or an animal model study is necessary to validate these findings.

This randomized controlled trial showed that UA has a modest effect on gut microbiota, such as increasing of Faith’s PD and *Ruminiclostridium* 5 in the 50 mg/day UA group. In addition, correlation analysis suggested that UA supplementation may be effective in UA non-producers. By analyzing the gut microbiota and its association with FMD improvement the results obtained have practical implications for a new approach to providing healthcare that focuses on intestinal environment-based diet therapy.

## Data availability statement

The datasets presented in this study can be found in online repositories. The names of the repository/repositories and accession number(s) can be found below: https://ddbj.nig.ac.jp/resource/sra-submission/DRA014918.

## Ethics statement

The studies involving human participants were reviewed and approved by Chiyoda Paramedical Care Clinic. The patients/participants provided their written informed consent to participate in this study.

## Author contributions

YN and KTF: visualization. YU and MK: methodology. YN: writing—original draft and formal analysis. YN, KTF, YU, MK, KZF, YK, and SF: writing—review and editing. All authors contributed to the article and approved the submitted version.

## Footnotes

1
https://center6.umin.ac.jp/cgi-open-bin/ctr_e/ctr_view.cgi?recptno=R000047949
